# Overwork

**Published:** 1871-09

**Authors:** 


### JOURNAL OF HEALTH.

VOL. XVIII. — SEPTEMBER, 1871. — NO. IX.

### OVERWORK.

A MOST mistaken notion is widely prevalent, that a good many people, especially brain workers, are killing themselves with hard study, and much misplaced sympathy is expended in their behalf. No man who gets a plenty of sleep was ever worked to death, either in body or brain. Hence, if a man of his own free will and choice deprives himself of necessary sleep, he is as much a suicide as if he starved himself to death. There may be a case now and then, although very rare, where the hours of sleep are deliberately curtailed to an unhealthful degree; but we may as well come to the real cause of maladies attributed to overwork: it is the unsystematic employment of time, and the unwise use of daily food — unwise in frequency, quantity, and quality.

The first great error is frequency. The majority of dyspeptics are made before twenty, on the part of girls especially, because they are always in the house, within easy reach of eatables of every description; these are more or less tempting, and few have the necessary force of character to resist with decision the inclinations of the appetite.

Physicians, as well as others, are liable to be hobby-horsical, but in sober judgment it is perhaps not too much to say, that if from the age of twelve years children were habituated, as a matter of principle and philosophy, to eat but three times a day, and nothing whatever between meals, more nervousness, more dyspepsia, more liver complaints, and more neuralgia would be prevented, than from the observance of all other rules together, about eating.

The mothers of young families are urged to possess themselves of the following facts, and impress them on the minds of their children on all suitable occasions, until firm root be taken in the thorough comprehension of their importance — facts which all educated physicians and physiologists of all schools, of all nations and both continents, admit and know to be true. The muscles of the body are the immediate cause of motion. Every part of the body which requires to be moved is supplied with one or more muscles. The movement of these muscles promotes health by aiding in the circulation of the blood.

Several parts of the body have two, three, or more muscles, and all must move in combination, in order to promote one motion. The heart has several muscles, so has the stomach, so has each limb. The beneficent Creator has so arranged it that necessary muscular motion is made without an effort in some cases, as in the heart, and in the motion of the eyelids; in other cases muscular motion is the cause of pleasurable sensations, as in walking out after several hours' confinement, as well as in effecting a kiss of one's sweetheart.

But all muscles may be moved too much, and they give evidence of tiredness in some cases, but not in all. It is not any trouble, requires no appreciable effort, to wink the eye or crack the finger; but, if either is attempted fifty times in quick succession, or a dozen times in quick succession, an appreciable effort is required; soon that effort becomes painful, and later on, impossible. So with walking; and when the limbs get tired, we stop and rest them. But some muscles are overworked without our knowing it; they do not complain in the way of tiredness, but in such a manner that the person does not know what is the matter, yet is conscious that something is wrong by the great amount of discomfort induced. A person eats too much, for example: a restlessness and discomfort takes hold of the whole system; this increases every moment until almost unendurable, when vomiting comes on, and when it is all over, the body returns to its usual healthfulness. The fact is, the stomach was tired; it was overworked, and could not perform its duty.

When a man eats a common meal, it requires the muscles of the stomach to work about five hours to put the food eaten into a condition for imparting nutriment to the body, and to be passed out of it. After this work, it requires rest; any man requires rest after five hours' work; but if, as soon as his task is done, another five hours' labor has been given, and is insisted upon, and so continued for several seasons of five hours' labor in any twenty-four, he would become so tired that he could not work at all. A child of six years of age can be made to feel the force of this argument. If a man eats a hearty meal five hours after breakfast, and another hearty meal five hours after dinner, and another five hours after supper, his stomach is kept working late into the night, while the body itself has long been resting; and if this is kept up, a premature loss of stomach power is inevitable, and a life-long dyspepsia, with all its daily horrors, repeated after each meal, is the inevitable result. Much sooner will these things follow, if a person habitually eats between meals, especially as this important fact is known: that when fresh food is taken into the stomach, before what was already there has been disposed of, this fresh food arrests the working up of what was there before, until it has been brought to the condition of that previously eaten; just as when water is boiling on the fire, if a lump of ice is thrown into it, the boiling ceases, and is not resumed until the ice has been melted and the water which it makes has been raised to the boiling point; and as the water would never boil if the lump of ice were constantly supplied, so the food is never digested, is never made to give strength to the body, if it is conveyed into the stomach at too frequent intervals. Hence, not giving strength, the dyspeptic is always weak, and the blind instinct, thinking, as it were, that food is wanting, is always crying for more. This explains why dyspeptics are always eating, are always hungry, and always weak. It is not meant to say that a grape or a melon, or a peach or orange, or apple or nice cake, or bit of candy or a delicious plate of ice-cream, should never be taken between meals, and that direful results would ensue from such individual infractions; on the contrary, for reasons not necessary to be named now, such things taken between meals now and then are of a positive advantage, if not taken over a day or two in succession; it is the daily, the persistent habit of such indulgences which causes the mischief, always, inevitably. Blessed the mother who makes it a matter of conscience and true parental love to inculcate these things on every child given to her.

